# Supplementary material for: A nutrient-responsive hormonal circuit mediates an inter-tissue program regulating metabolic homeostasis in adult Drosophila
Source: Nat Commun. 2021 Aug 30;12:5178. doi: 10.1038/s41467-021-25445-2 (PMC8405823; doi:10.1038/s41467-021-25445-2)
Supplement: Supplementary file 1 — Supplementary Information [file 41467_2021_25445_MOESM1_ESM.pdf]

SUPPLEMENTARY INFORMATION

A nutrient-responsive hormonal circuit mediates an inter-tissue program regulating metabolic homeostasis in adult *Drosophila*

Takashi Koyama[1,†], Selim Terhzaz[2, 3,†], Muhammad T. Naseem[1], Stanislav Nagy[1], Kim Rewitz[1], Julian A. T. Dow[2], Shireen A. Davies[2] and Kenneth V. Halberg[1,*]

[1] Section for Cell and Neurobiology, Department of Biology, University of Copenhagen, Universitetsparken 15, DK-2100 Copenhagen, Denmark
[2] Institute of Molecular, Cell and Systems Biology, College of Medical, Veterinary and Life Sciences, University of Glasgow, Glasgow G12 8QQ, UK
[3] MRC-University of Glasgow Centre for Virus Research, Glasgow G61 1QH, Scotland, UK.

[†], Authors contributed equally
[*]Author for correspondence: kahalberg@bio.ku.dk

Contains:
Supplementary Figures 1-8
Supplementary Tables 1-2

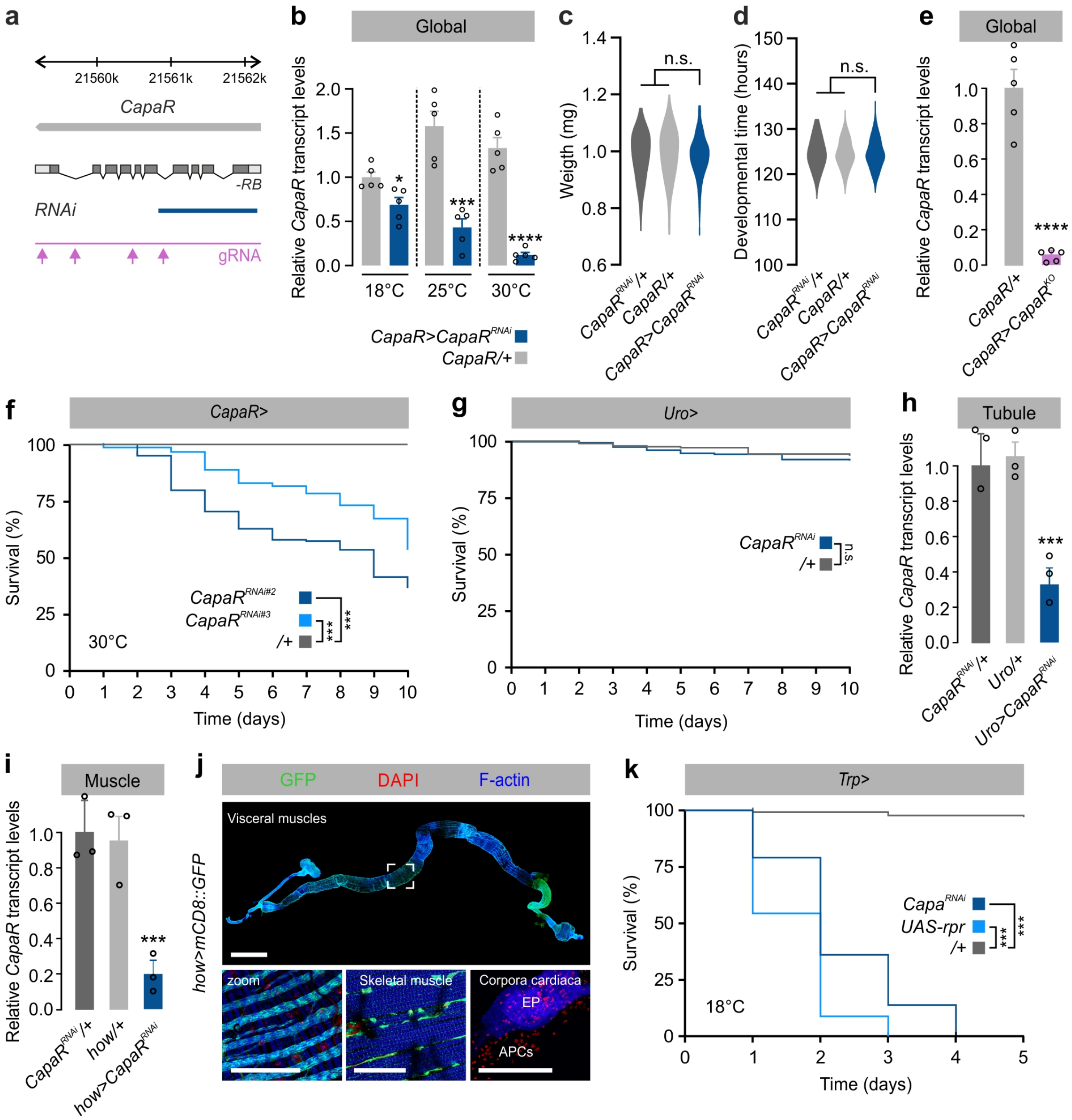

Supplementary Figure 1. Extra-renal Capa/CapaR signaling is essential for adult fly viability. a. Exon map of the *CapaR* gene indicating the regions targeted by RNAi (blue; in-house generated doubly homozygous 2xRNAi line) and CRISPR/Cas9-mediated (magenta) genome editing techniques. **b.** *CapaR* transcript abundance normalized to *RpL3* following global 2xRNAi-mediated *CapaR* knockdown in flies raised at 18°C (*P*=0.150), 25°C (*P*=0.0004) and 30°C (*P*<0.0001) (mean ± SEM; one-way ANOVA; n=5). **c.** Pupal weight of global *CapaR* knockdown flies (*CapaR>CapaR^{RNAi}*; n=134) throughout development is not significantly different compared to *CapaR*>+ (n=111) and C*apaR^{RNAi}*/+ (n=140) parental controls (one-way ANOVA; n.s. *P*>0.05). **d.** Developmental time of global *CapaR* knockdown flies (*CapaR>CapaR^{RNAi}*; n=129) throughout development is not significantly different compared to *CapaR*>+ (n=107) and C*apaR^{RNAi}*/+ (n=123) parental controls (one-way ANOVA). **e.** *CapaR* transcript abundance normalized to *RpL3* following global *CapaR* knockout (mean ± SEM; two-tailed Student's *t*-test; *P*>0.0001; n=5). **f.** Two independent

RNAi lines, *CapaR*$^{RNAi\#2}$ (n=183) and *CapaR*$^{RNAi\#3}$ (n=151), show the same mortality phenotype relative to control (*CapaR*>+; n=151) confirming the specificity of the RNAi-effect (two-sided log-rank test; *P*<0.001). **g.** Knockdown of *CapaR* exclusively in tubule principal cells (n= 287) did not induce significant changes in fly survival compared to control (*Uro*>+; two-sided log-rank test; n=231). **h-i.** *CapaR* transcript abundance normalized to *RpL32* following 2xRNAi-mediated *CapaR* knockdown in tubules or muscles (mean ± SEM; one-way ANOVA; *P*<0.001; n=5). **j.** Analyzing *how>mCD8::GFP* (green) confirm GAL4-driven expression in visceral and skeletal muscles, but not the APCs, of adult *Drosophila*. The experiment was repeated twice with the same results. Scale bar 75 μm. **k.** Knocking down *Capa* expression (n=72) or completely ablating (n=70) the Capa-producing neurons by ectopic expression of *rpr*, phenocopies the mortality of *CapaR* silencing in adult *Drosophila* relative to control (*Trp*>+; two-sided log-rank test; *P*<0.001; n=133).

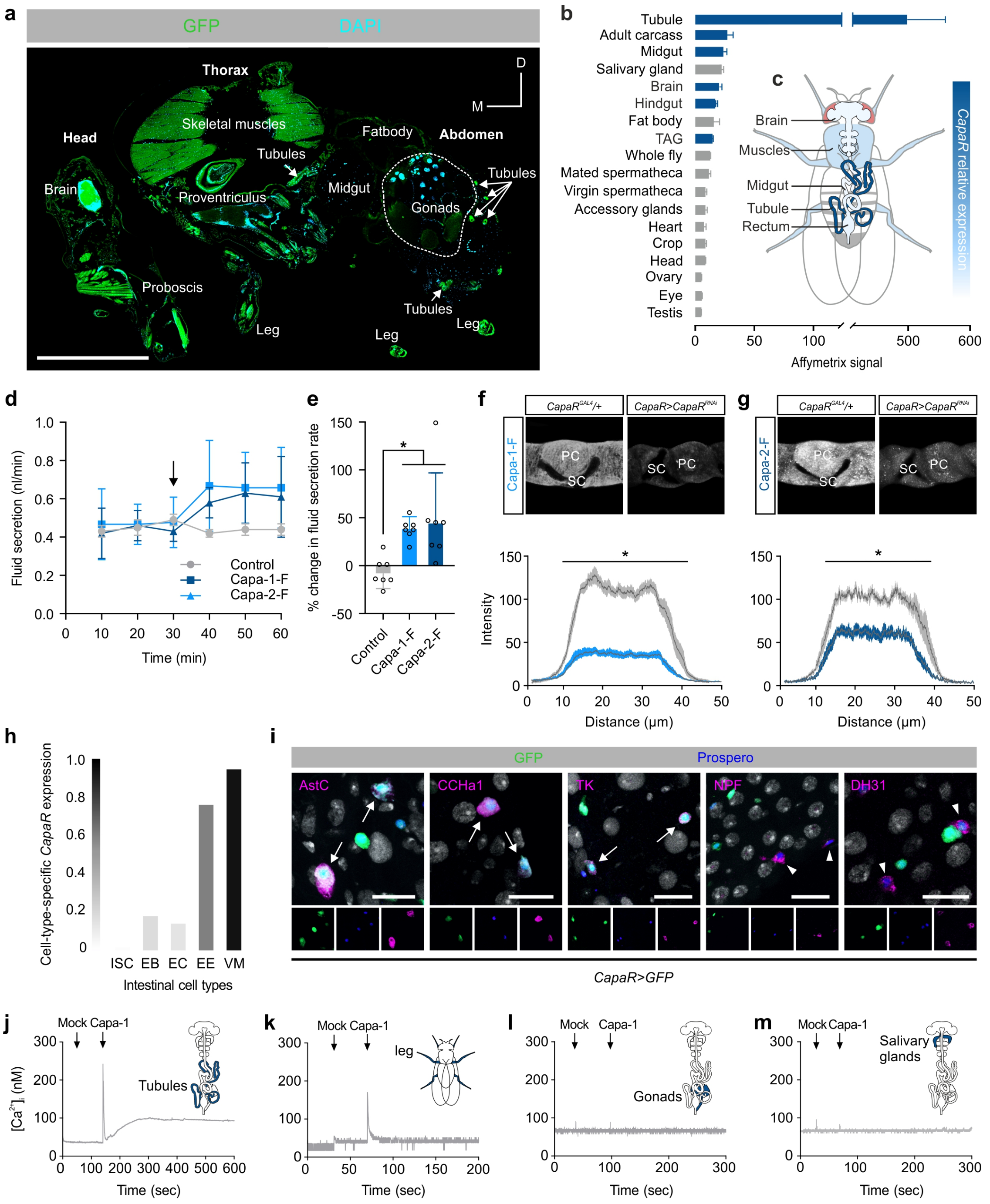

Supplementary Figure 2. Spatial expression analysis and functional validation of CapaR. a. Medial paraffin section of adult *CapaR>mCD8::GFP* fly showing GFP expression in different tissues, including the tubules, brain, somatic musculature, and gut. This experiment was performed three times with similar results. Scale bar = 0.5 mm. **b.** Mean normalized Affymetrix signal ± SEM showing *CapaR* spatial expression across major adult tissues. **c.** Relative *CapaR* expression superimposed on an anatomical map of adult *Drosophila*. **d-e.** Validation of fluorophore-coupled Capa-1 (Capa-1-F) and Capa-2 (Capa-2-F) peptides. **d.** Fluid secretion assays show significant functional stimulation of renal tissues

by Capa-1-F and Capa-2-F peptides demonstrating biological activity. **e.** Percent change in fluid secretion rate following addition of Capa-1-F or Capa-2-F ligands (mean ± SEM; one-way ANOVA; $P<0.05$; n= 7). **f-g.** Knockdown of *CapaR* expression significantly reduces fluorescent intensity in principal cells compared to parental controls following **f.** Capa-1-F and **g.** Capa-2-F application (mean ± SEM; one-way ANOVA; $P<0.05$; n=5). **h.** Relative expression of *CapaR* in the different cell types of the intestine as realized by FACS sorted cell populations. Intestinal stem cells, ISC; Enteroblasts, EB; Enterocytes, EC; Enteroendocrine cells, EEs; Visceral muscles, VM. Data from: flygutseq.buchonlab.com[1]. **i.** The transcription factor Prospero (blue) colocalizes with *CapaR*>-driven *mCD8::GFP* expression (green) as well as the peptides Allatostatin C (AstC), CCHamide1 (CCHa1) and Tachykinin (TK) but not Neuropeptide F (NPF) or Diuretic Hormone 31 (DH31) (magenta). The experiment was repeated several times with the same results. Scale bar 10 µm. **j.-m.** Functional validation of tissue-specific Capa/CapaR signaling using *in vivo* calcium-reporter technology. Stereotypic biphasic increases in cytosolic $[Ca^{2+}]_i$ was detected upon Capa-1 application (10^{-7} M) in **j.** tubules, and **k.** legs, but not in **l.** testes or **m.** salivary glands.

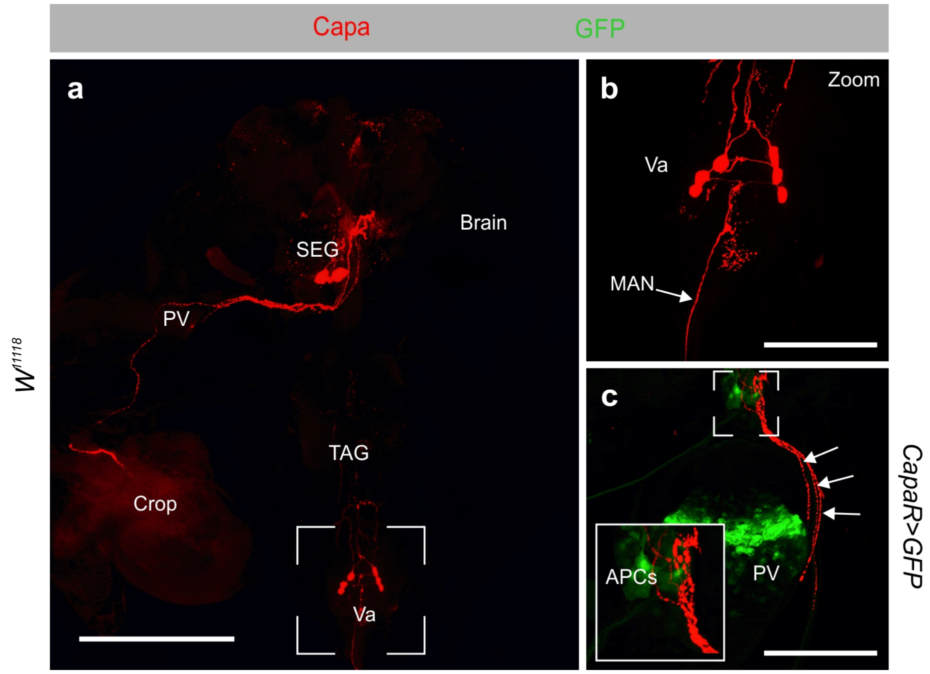

Supplementary Figure 3. Neuroanatomy of Capa⁺ neurons. a. Immunoreactivity of Capa precursor antibody (red) identifies one pair of SEG neurons and three pairs of Va Capa⁺ neurons. The experiment was repeated multiple times with the same results. Scale bar = 200 µm. **b.** The Va neurons release Capa peptides into circulation via the MAN. Scale bar = 100 µm. **c.** The axons from the SEMG neurons innervate the APCs and PV, which also shows *CapaR>mCD8::GFP* expression (green) in muscle cells. Scale bar = 100 µm.

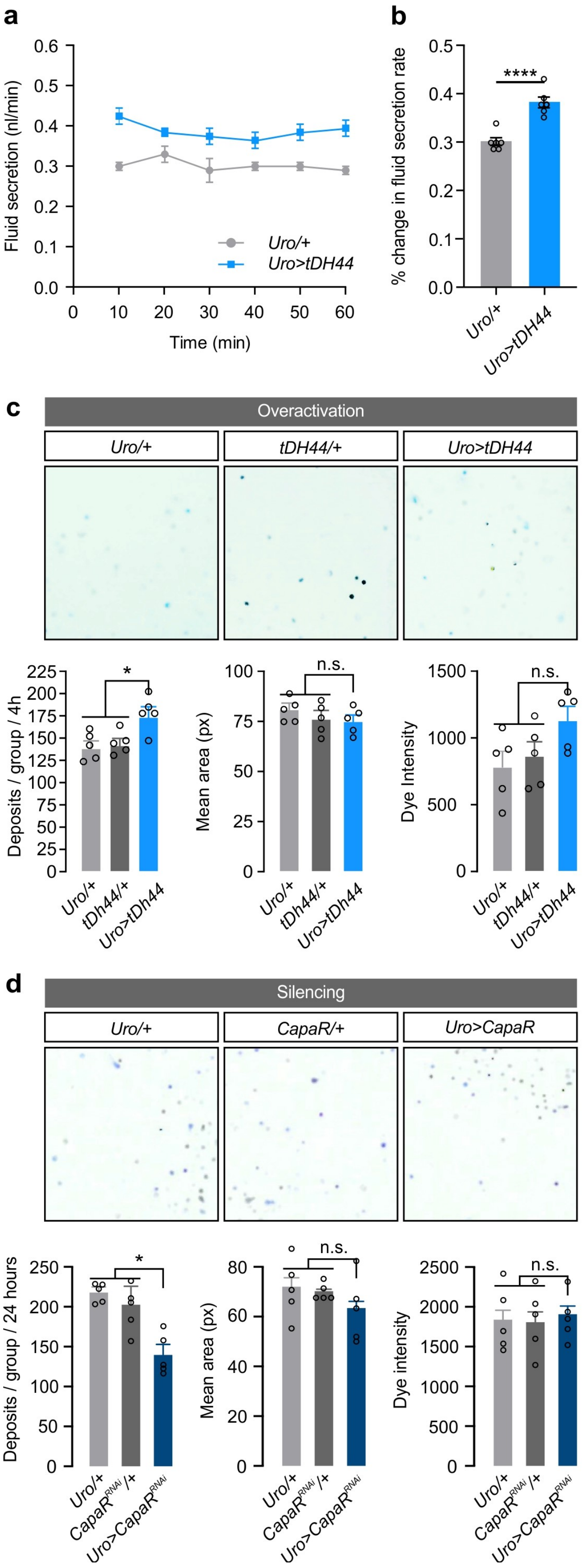

Supplementary Figure 4. Manipulating renal tubule secretion augments fecal output characteristics. a. Fluid secretion assays show elevated basal rates of urine production in tubules with constitutive activation of the DH44 pathway (*Uro>tDH44*) relative to control (*Uro/+*). **b.** The basal rate of secretion is significantly elevated in *Uro>tDH44* flies compared to control (*Uro/+*; two-tailed unpaired Student's *t*-test; mean ± SEM; *P*<0.0001; n=6). **c.** Fecal output profiles following DH44 activation in the tubules (*Uro>tDH44*) reveal that these flies produce more, but not larger of more dilute, deposits compared to controls (mean ± SEM; one-way ANOVA; *P*<0.05; n=5). **d.** Fecal output profiles following *CapaR* silencing in the tubules (*Uro>CapaR*) indicate that these flies produce a similar number, albeit smaller and more concentrated, deposits compared to controls (one-way ANOVA; mean ± SEM; *P*<0.05; n=5).

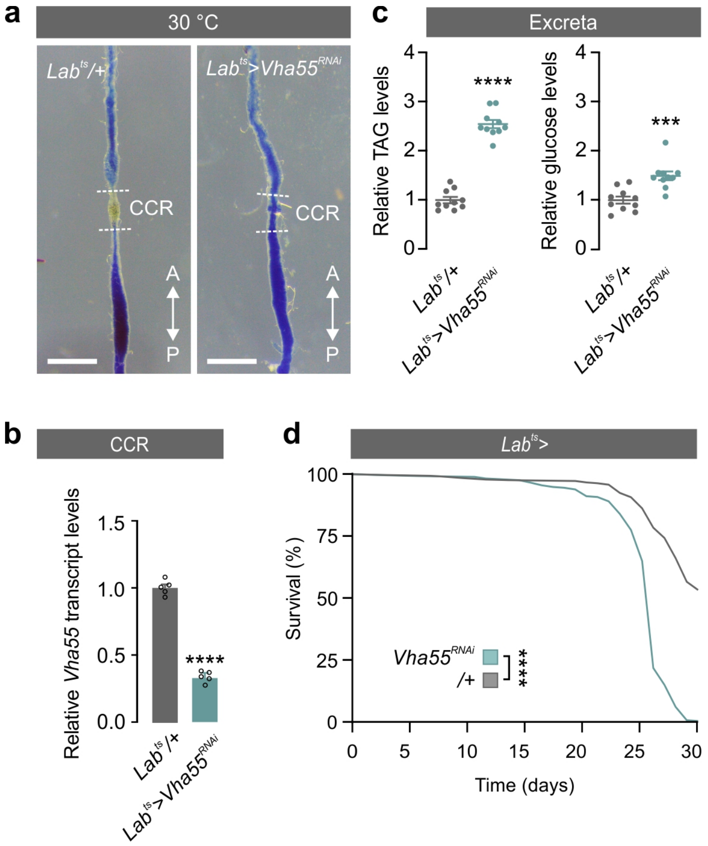

Supplementary Figure 5. Loss of CCR acidity impairs nutrient absorption and adult fly viability. a. Acute knockdown of *Vha55* specifically in the CCR (Lab^{ts}>$Vha55^{RNAi}$) reduces midgut acidity. The experiment was repeated twice with the same results. **b.** *Vha55* transcript abundance normalized to *RpL3* following CCR-specific *Vha55* knockdown (mean ± SEM; two-tailed Student's *t*-test; P<0.0001; n=5). **c.** Relative amounts of undigested triacylglyceride (TAG; P<0.0001) and glucose levels (P<0.0004) in excreta from control (Lab^{ts}/+) and knockdown (Lab^{ts}>$Vha55^{RNAi}$) flies raised on standard medium (mean ± SEM; two-tailed Student's *t*-test; n=10)**. d.** Knockdown of *Vha55* in the CCR (Lab^{ts}>$Vha55^{RNAi}$; n=293) causes significant fly mortality compared to control (Lab^{ts}/+; two-sided log-rank test; P<0.0001; n=333).

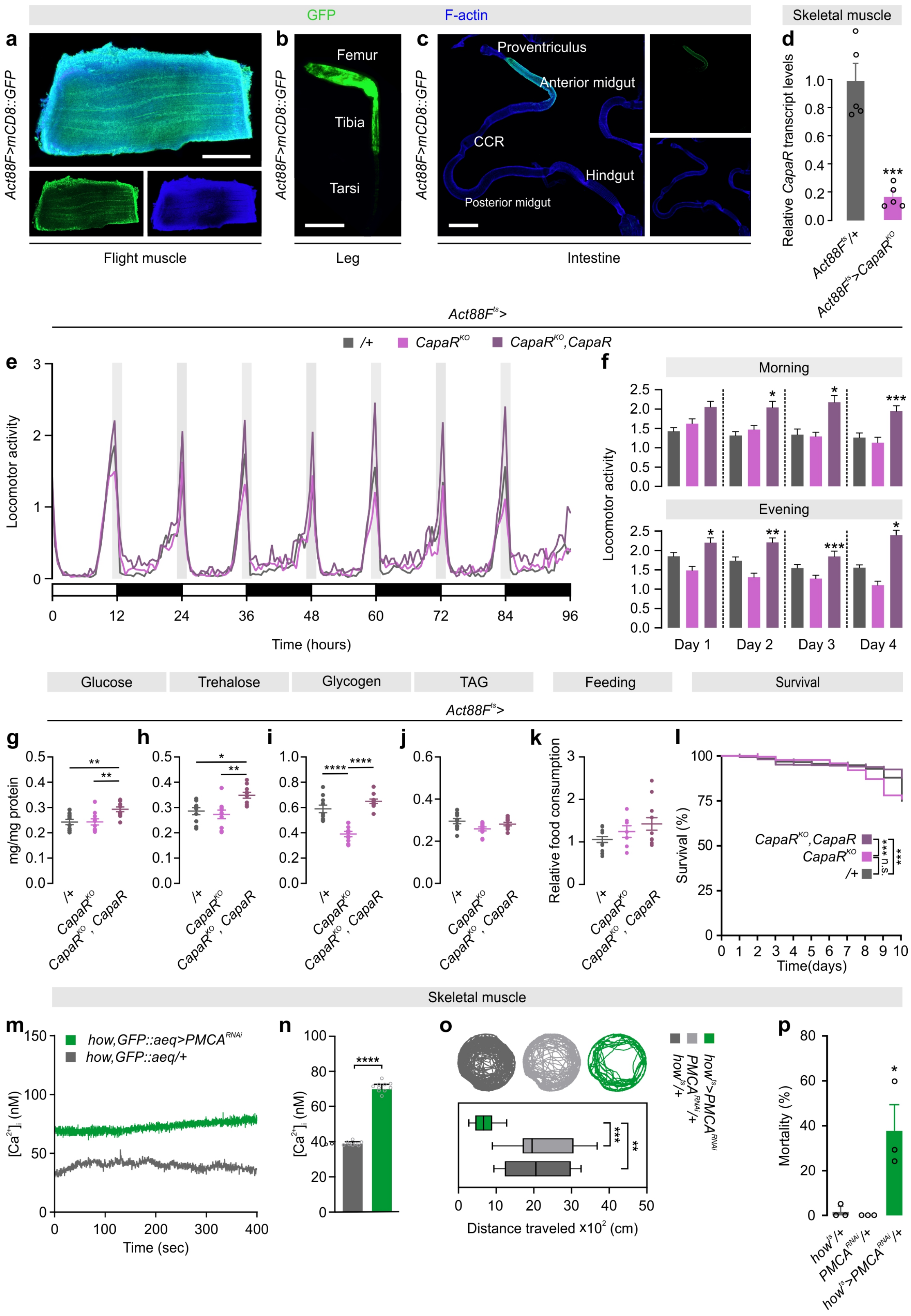

GFP
F-actin
Skeletal muscle
a
b
c
d
Act88F>mCD8::GFP
Flight muscle
Femur
Tibia
Tarsi
Leg
Proventriculus
Anterior midgut
CCR
Hindgut
Posterior midgut
Intestine
Relative CapaR transcript levels
Act88F^ts/+
Act88F^ts>CapaR^KO
Act88F^ts>
/+
CapaR^KO
CapaR^KO,CapaR
e
f
Locomotor activity
Time (hours)
Morning
Evening
Day 1
Day 2
Day 3
Day 4
Glucose
Trehalose
Glycogen
TAG
Feeding
Survival
g
h
i
j
k
l
mg/mg protein
Relative food consumption
Survival (%)
Time(days)
n.s.
Skeletal muscle
m
n
o
p
how,GFP::aeq>PMCA^RNAi
how,GFP::aeq/+
[Ca2+]i (nM)
Time (sec)
how^ts>PMCA^RNAi
PMCA^RNAi/+
how^ts/+
Distance traveled x10^2 (cm)
Mortality (%)

Supplementary Figure 6. Physiological effects of eliminating Capa/CapaR signaling in skeletal muscles. a-c. Immunofluorescence of tissues from *Act88F>mCD8::GFP* flies counter-stained with Phalloidin showed an overlap between GFP (green) and F-actin (blue) immunoreactivity in the flight muscles, legs and in the visceral muscles of the anterior part of the gut. The gut staining does not overlap with *CapaR>mCD8::GFP* expression. The experiment was repeated twice with the same results. Scale bar = 200 µm. **d.** *CapaR* transcript abundance normalized to *RpL32* following *CapaR* knockout using *Act88F-Gal4* (mean ± SEM; two-tailed Student's *t*-test; *P*<0.001; n=5). **e.** Locomotor activity of individual control ($Act88F^{ts}$/+; n=31), skeletal muscle-specific *CapaR* knockout ($Act88F^{ts}>CapaR^{KO}$; n=31) and rescue ($Act88F^{ts}>CapaR^{KO}$,*CapaR*; n=31) flies exposed to 12-hour:12-hour, light-dark (LD) cycles over 96 hours. **f.** Morning and evening activity peaks measured during the experimental period (mean ± SEM; one-way ANOVA; *, *P*<0.05; **, *P*<0.01; ***, *P*<0.001). **g-j.** Quantification of **c.** glucose, **d.** trehalose, **e.** glycogen and **f.** TAG levels in flies with the different genotypes (mean ± SEM; one-way ANOVA; *, *P*<0.05; **, *P*<0.01; ****, *P*<0.0001; n=10). **k.** Relative food intake (mean ± SEM; one-way ANOVA; n=10). **l.** Kaplan-Meier survival curves of control (/+; n=508), knockout ($Act88F^{ts}>CapaR^{KO}$; n=594) and rescue ($Act88F^{ts}>CapaR^{KO}$,*CapaR*; n=677) flies (two-sided log-rank test; *P*<0.001). **m-n.** Quantification of $[Ca^{2+}]_i$ following *PMCA* knockdown in skeletal muscles (*how, GFP::aeq*>$PMCA^{RNAi}$) relative to control (*how,GFP::aeq*/+; mean ± SEM; two-tailed Student's *t*-test; *P*<0.0001; n=3). **o.** Representative activity traces of video-tracked individual flies with targeted *PMCA* knockdown in muscles ($how^{ts}>PMCA^{RNAi}$) relative to control flies, including quantification of distance travelled (Tukey's box-plots; whiskers: min and max; box: 25th and 75th percent quartiles; middle: median; one-way ANOVA; **, *P*<0.01; ***, *P*<0.001; n=9). **p.** Knockdown of *PMCA* in muscles ($how^{ts}>PMCA^{RNAi}$) causes significant fly mortality (mean ± SEM; one-way-ANOVA; *P*<0.05; n=3 biologically independent replicates with 20 flies per replicate).

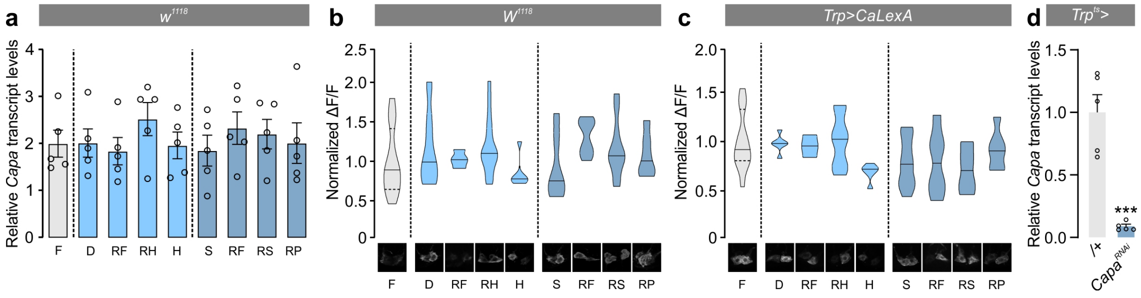

Supplementary Figure 7. Capa^{+} SEG neuron activity is unaffected by water or nutrient stress. a. Transcript levels of *Capa* relative to *RpL3* in the SEG neurons (head samples) from flies exposed to different environmental conditions (mean ± SEM; one-way ANOVA; n=5). **b.** Violin plots of immunofluorescence quantifications of intracellular Capa precursor levels (n=6-16) and **c.** CaLexA-induced GFP expression in Capa-producing SEG neurons using *Trp-GAL4* (n=4-12) from flies exposed to different environmental conditions (one-way ANOVA). F, fed; D, desiccated; RF, refed; RH, rehydrated; H, hydrated; S, starved; RS, refed sugar; RP, refed protein. **d.** *Capa* transcript abundance (thorax samples) normalized to *RpL32* following *Capa* knockdown using *Trp^{ts}* (mean ± SEM; two-tailed Student's *t*-test; *P*<0.001; n=5).

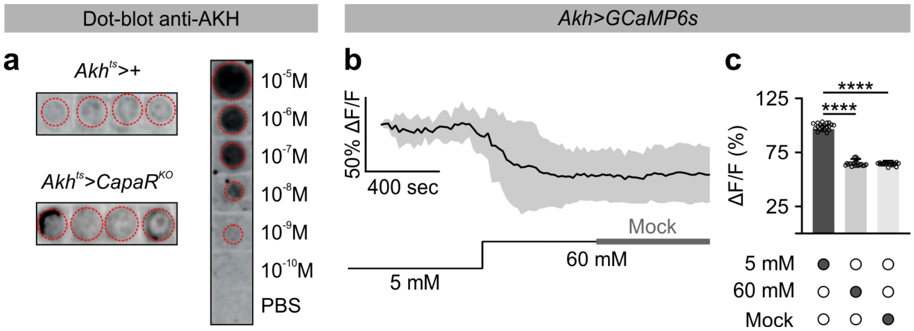

Supplementary Figure 8. Anti-AKH dot-blots and CC excitability by mock perfusion. a. Dot-blot source data of hemolymph samples from control (*Akh*^{ts}>+) and *CapaR* depleted animals (*Akh*^{ts}>C*apaR*^{KO}) as well as known concentrations of AKH (standards). **b.** ΔF/F traces (mean ± SEM) of CCs from flies expressing the GCaMP6s calcium sensor (*Akh>GCaMP6s*) sequentially responding to low sugar (5mM trehalose), high sugar (60mM trehalose) and high sugar without Capa-1 (= mock). **c.** ΔF/F of CCs responding (mean ± SEM) to sugar increases and mock perfusion (mean ± SEM; one-way ANOVA; *P*<0.0001; n=7).

Supplementary Table S1. Primer sequences used in *Drosophila* transgenesis.

	**Primer sequence**
NotI-CapaR_F	5'-CGCGGCCGCATGAATTCATCGACCGATCCGAC-3'
KpnI-CapaR_R	5'-GCGGTACCTTAAATACAAGTCTCCTCGTTCTCGATCC-3'
CapaR_CRISPR1_F	5'-CGGCCCGGGTTCGATTCCCGGCCGATGCAGCTGTTGGATGGTGAAG TTCGTTTCAGAGCTATGCTGGAAAC-3'
CapaR_CRISPR1_R	5'-AATCAATCCCAACAGAATCCTGCACCAGCCGGGAATCGAACC-3'
CapaR_CRISPR2_F	5'-GGATTCTGTTGGGATTGATTGTTTCAGAGCTATGCTGGAAAC-3'
CapaR_CRISPR2_R	5'-GCTTGTATTCCACGTGTGTATGCACCAGCCGGGAATCGAACC-3'
CapaR_CRISPR3_F	5'-TACACACGTGGAATACAAGCGTTTCAGAGCTATGCTGGAAAC-3'
CapaR_CRISPR3_R	5'-ATTTTAACTTGCTATTTCTAGCTCTAAAACAGTCATATCACTAAATT CCCTGCACCAGCCGGGAATCGAACC-3'

Supplementary Table S2. Quantitative RT-PCR primers used.

**Primer name**	**Primer sequence**
CapaR_F	5'-TACCCCAGATACCCTATTCGCC-3'
CapaR_R	5'-CTAAGAGATTACCAACGACCCCG-3'
GlyS_F	5'-GCAAGCTGTTTACCCAGACTATGTGG-3'
GlyS_R	5'-CAATTCCTTGAGTTCGGTCTCCTC-3'
AGBE_F	5'-CGGCGATTACAACGAGTACTTTGG-3'
AGBE_R	5'-GGTAGGCATTCCAGAAACATCCTC-3'
GlyP_F	5'-AGTACGACCACTACTACTTGCTGG-3'
GlyP_R	5'-CGCTGGACGCAATGTTGTTGATGG-3'
AGL_F	5'-GATTCAGTCCTGCTCTAAGTCCAC-3'
AGL_R	5'-CACGCAGAGCGATGAAGGTATCAC-3'
Akh_ F	5'-TCCCAAGAGCGAAGTCCTCA-3'
Akh_R	5'-GTCCAGAAAGAGCTGTGCCT-3'
PMCA_F	5'-TCTATTGCTCCGCAAACCCTATGG-3'
PMCA_R	5'-GTCCACGTCCAGATTCAATATCGAG-3
Vha55_F	5'-CCCGCTTGACTTACAAGACTGTGTC-3'
Vha55_R	5'-AATGTCGCCGGTGAACTCGCAAAG-3'
Capa_F	5'-ATTTACGGAGATGCCTCCCAGGAG-3'
Capa_R	5'-TTCCTTCTGTCCCTTGGAGATGTCC-3
RpL32_F	5'-TGACCATCCGCCCAGCATAC-3'
RpL32_R	5'-ATCTCGCCGCAGTAAACG-3'
RpL3_F	5'-AAGGATGACGCCAGCAAGCCAGTC-3'
RpL3_R	5'-TAGCCGACAGCACCGACCACAATC-3'

References

1 Dutta, D. *et al.* Regional Cell-Specific Transcriptome Mapping Reveals Regulatory Complexity in the Adult Drosophila Midgut. *Cell reports* **12**, 346-358, doi:10.1016/j.celrep.2015.06.009 (2015).
