# Supplementary material for: A nutrient-responsive hormonal circuit mediates an inter-tissue program regulating metabolic homeostasis in adult Drosophila
Source: Nat Commun. 2021 Aug 30;12:5178. doi: 10.1038/s41467-021-25445-2 (PMC8405823; doi:10.1038/s41467-021-25445-2)
Supplement: Supplementary file 3 — Reporting Summary [file 41467_2021_25445_MOESM3_ESM.pdf]

nature research

Corresponding author(s): Kenneth V. Halberg

Last updated by author(s): Jul 2, 2021

Reporting Summary

Nature Research wishes to improve the reproducibility of the work that we publish. This form provides structure for consistency and transparency in reporting. For further information on Nature Research policies, see our Editorial Policies and the Editorial Policy Checklist.

Statistics

For all statistical analyses, confirm that the following items are present in the figure legend, table legend, main text, or Methods section.

n/a	Confirmed	
☐	☒	The exact sample size (n) for each experimental group/condition, given as a discrete number and unit of measurement
☐	☒	A statement on whether measurements were taken from distinct samples or whether the same sample was measured repeatedly
☐	☒	The statistical test(s) used AND whether they are one- or two-sided<br>*Only common tests should be described solely by name; describe more complex techniques in the Methods section.*
☒	☐	A description of all covariates tested
☐	☒	A description of any assumptions or corrections, such as tests of normality and adjustment for multiple comparisons
☐	☒	A full description of the statistical parameters including central tendency (e.g. means) or other basic estimates (e.g. regression coefficient) AND variation (e.g. standard deviation) or associated estimates of uncertainty (e.g. confidence intervals)
☐	☒	For null hypothesis testing, the test statistic (e.g. F, t, r) with confidence intervals, effect sizes, degrees of freedom and P value noted<br>*Give P values as exact values whenever suitable.*
☒	☐	For Bayesian analysis, information on the choice of priors and Markov chain Monte Carlo settings
☒	☐	For hierarchical and complex designs, identification of the appropriate level for tests and full reporting of outcomes
☒	☐	Estimates of effect sizes (e.g. Cohen's d, Pearson's r), indicating how they were calculated

Our web collection on statistics for biologists contains articles on many of the points above.

Software and code

Policy information about availability of computer code

Data collection	We did not use any unpublished code to collect the data in this study. We used LSM900 and ZEN software (Carl Zeiss, ZEN, Blue edition) for image acquisition. AZtecEnergy microanalysis software version (Oxford instruments, Oxford, UK) was used for element microanalysis quantification.
Data analysis	We did not use any unpublished code to analyze the data in this study. We used ZEM image analyzing software (Carl Zeiss, ZEN, Blue edition), Graphpad prism 9.1, ImageJ (Fiji, 2.1.0), Microsoft Excel (Microsoft office professional Plus 2016), T.U.R.D - the ultimate reader of dung (version 0.8), and AZtecEnergy microanalysis software version (Oxford instruments, Oxford, UK) was used for data analyses.

For manuscripts utilizing custom algorithms or software that are central to the research but not yet described in published literature, software must be made available to editors and reviewers. We strongly encourage code deposition in a community repository (e.g. GitHub). See the Nature Research guidelines for submitting code & software for further information.

Data

Policy information about availability of data

All manuscripts must include a data availability statement. This statement should provide the following information, where applicable:

- Accession codes, unique identifiers, or web links for publicly available datasets
- A list of figures that have associated raw data
- A description of any restrictions on data availability

All raw data are available from the corresponding author on reasonable request. Source data underlying the figures presented are provided.

Field-specific reporting

Please select the one below that is the best fit for your research. If you are not sure, read the appropriate sections before making your selection.

☒ Life sciences ☐ Behavioural & social sciences ☐ Ecological, evolutionary & environmental sciences

For a reference copy of the document with all sections, see nature.com/documents/nr-reporting-summary-flat.pdf

Life sciences study design

All studies must disclose on these points even when the disclosure is negative.

Sample size	No sample size was decided before the experiments. Sample size was determined based on the consistency of measurable differences between groups. We used our previously published work (e.g. Koyama et al., 2021, PNAS) to determine the statistical methods.
Data exclusions	We did not exclude any data in this study.
Replication	We replicated all the experiments and compared the data at least twice independently. All attempts of replication was successful.
Randomization	We randomized the population of flies within the same genotype for each experiement. We matched the sex and age of flies in every trial.
Blinding	To limit bias, investigators checked genotypes after conducting the experiments and data collections. Key experiments were carried by multiple authors.

Reporting for specific materials, systems and methods

We require information from authors about some types of materials, experimental systems and methods used in many studies. Here, indicate whether each material, system or method listed is relevant to your study. If you are not sure if a list item applies to your research, read the appropriate section before selecting a response.

Materials & experimental systems

n/a	Involved in the study	
☐	☒	Antibodies
☒	☐	Eukaryotic cell lines
☒	☐	Palaeontology and archaeology
☐	☒	Animals and other organisms
☒	☐	Human research participants
☒	☐	Clinical data
☒	☐	Dual use research of concern

Methods

n/a	Involved in the study	
☒	☐	ChIP-seq
☒	☐	Flow cytometry
☒	☐	MRI-based neuroimaging

Antibodies

Antibodies used	The primary antibodies used are as follows: rabbit anti-CapaR (1:500; a generous gift from Shireen A.-Davies, University of Glasgow, UK), rabbit anti-Capa precursor peptide (1:500; a gift from Jan Veenstra, Université de Bordeaux, France), rabbit anti-AstC, rabbit anti-CCHa1, rabbit anti-DH31, rabbit anti-NPF, rabbit anti-TK (1:100; gifts from Jan Veenstra, Université de Bordeaux, France) and rabbit anti-AKH (1:500; a generous gift from Dr. Jae Park, University of Tennessee, US). Detailed information on antibody design and production including any commercial sources are listed in the references provided for each antibody used in the manuscript as well as in the validation section below. Additional commercial primary antibodies used were mouse anti-Prospero (1:50; Developmental Studies Hybridoma bank, AB_528440), DyLight 488 conjugated goat anti-GFP (1:500, ThermoFisher, 600-141-215), and Alexa Fluor 488-conjugated mouse anti-GFP (1:500, Invitrogen, A11120). The primary antibodies were visualized with: goat anti-rabbit or goat anti-mouse Alexa Fluor 488, 555 or 594 (1:500; ThermoFischer #A32731/#A32723, #A21429 or #R37117). For Dot-blot assay, goat anti-rabbit IRDye 800CW (1:2500; LI-COR, 926-32211) was used.
Validation	All the primary antibodies used in this study were confirmed in the previous studies as listed below:<br>1. Alexa Fluor 488-conjugated mouse anti-GFP and DyLight 488 conjugated goat anti-GFP: Halberg, K. A., Terhzaz, S., Cabrero, P., Davies, S. A. & Dow, J. A. Tracing the evolutionary origins of insect renal function. Nat Commun 6, 6800, (2015).<br>2. Rabbit anti-CapaR: Terhzaz, S. et al. Mechanism and function of Drosophila capa GPCR: a desiccation stress-responsive receptor with functional homology to human neuromedinU receptor. PLoS One 7, e29897 (2012).<br>3. Rabbit anti-Capa precursor peptide: Kean, L. et al. Two nitridergic peptides are encoded by the gene capability in Drosophila melanogaster. American journal of physiology. Regulatory, integrative and comparative physiology 282, R1297-1307 (2002).<br>4.-8. Rabbit anti-AstC; Rabbit anti-CCHa1; Rabbit anti-DH31; Rabbit anti-NPF; Rabbit anti-TK: Veenstra, J. A. & Ida, T. More Drosophila enteroendocrine peptides: Orcokinin B and the CCHamides 1 and 2. Cell and tissue research 357, 607-621 (2014). Veenstra, J. A., Agricola, H. J. & Sellami, A. Regulatory peptides in fruit fly midgut. Cell and tissue research 334, 499-516,<br>9. Mouse anti-Prospero: Evidence that stem cells reside in the adult Drosophila midgut epithelium. Nature 439, 475-479, (2006).

10. Rabbit anti-AKH: Lee, G. & Park, J. H. Hemolymph sugar homeostasis and starvation-induced hyperactivity affected by genetic manipulations of the adipokinetic hormone-encoding gene in Drosophila melanogaster. Genetics 167, 311-323.

Animals and other organisms

Policy information about studies involving animals; ARRIVE guidelines recommended for reporting animal research

Laboratory animals	Male flies (Drosophila melanogaster) 4 day-old (unless otherwise stated) at the start of experimentation were used in this study.
Wild animals	We did not use wild animals in this study.
Field-collected samples	We did not use any field-collected samples or animals in this study.
Ethics oversight	No ethical approval was required for this study.

Note that full information on the approval of the study protocol must also be provided in the manuscript.
